# Pathogen-targeted quick multiplex PCR identification of *Chlamydia psittaci* and *Haemophilus influenzae* coinfection in pneumonia: a case report

**DOI:** 10.3389/fmed.2026.1890644

**Published:** 2026-10-05

**Authors:** Ying Yuan, Jianhong Wang, Ning Feng, Fen Wu, Xiao Dong, Youxin Xu, Shunfeng Zhao, Tingzhao Tan

**Affiliations:** 1 Department of Clinical Laboratory, Liaocheng Third People’s Hospital, Liaocheng, China; 2 Department of Critical Care Medicine, Liaocheng Third People’s Hospital, Liaocheng, China; 3 Department of Medical Oncology, Liaocheng Tumor Hospital, Liaocheng, China

**Keywords:** case report, *Chlamydia psittaci*, *Haemophilus influenzae*, metagenomic next-generation sequencing, pt-qPCR, rapid molecular diagnosis

## Abstract

This study describes a rare case of pneumonia induced by coinfection with *Chlamydia psittaci* (*C. psittaci*) and *Haemophilus influenzae (H. influenzae)*. The patient had a history of chronic nephritis and long-term pigeon breeding. The initial manifestation was slurred speech, followed by rapid progression to high fever and respiratory failure. Chest computed tomography revealed bilateral interstitial pneumonia, predominantly in the right lung. Pathogen-targeted quick multiplex polymerase chain reaction (pt-qPCR) confirmed the pathogens as *C. psittaci* and *H. influenzae*, and metagenomic next-generation sequencing (mNGS) further verified consistent results. After 7-days of combination therapy with doxycycline and azithromycin, the treatment was switched to doxycycline combined with moxifloxacin for an additional 8-days. The patient achieved complete recovery and was discharged from the hospital. This case suggests that pt-qPCR may facilitate early identification of mixed infections in patients with severe pneumonia and a history of avian exposure, thereby providing valuable information to guide clinical decision-making.

## 1 Introduction

Psittacosis is a rare zoonotic infectious disease with highly variable clinical features, which is caused by *Chlamydia psittaci* (*C. psittaci*) (1). The disease was first reported in 1879, indicating an association between avian exposure and fatal respiratory diseases in humans (2). *C. psittaci* infects humans mainly through respiratory inhalation of airborne droplets and aerosols, or direct contact with avian excreta, thereby causing community-acquired pneumonia (CAP) (3). Epidemiological data show that *C. psittaci* accounts for approximately 1% of CAP cases in China, with a detection rate of up to 8.0% in severe CAP (4). The clinical manifestations of this bacterial infection are non-specific; its common symptoms, including high fever, cough, and dyspnea, are similar to those of common respiratory infections, making it prone to misdiagnosis and missed diagnosis (4, 5). In some cases, the disease progresses rapidly, leading to severe organ damage such as an Acute Respiratory Distress Syndrome (ARDS) and shock, involving multiple systems. *Haemophilus influenzae* (*H. influenzae*) is a pleomorphic Gram-negative coccobacillus and one of the most common pathogens causing acute and chronic lower respiratory tract infections. Due to the atypical clinical manifestations and difficult diagnosis of psittacosis, severe pneumonia caused by *C. psittaci* complicated with *H. influenzae* infection is highly prone to missed diagnosis and rarely reported. However, given its potential to induce severe infection, rapid clinical progression and even life-threatening complications, achieving timely and accurate etiological diagnosis is of critical importance to halt the deterioration of this mixed infection.

The diagnosis of this mixed infection relies on comprehensive evaluation integrating clinical manifestations, epidemiological exposure history and etiological findings. Conventional etiological detection approaches still have inherent limitations. In comparison with routine culture-based methods, molecular detection techniques feature higher sensitivity and shorter turnaround time. Pathogen-targeted quick multiplex polymerase chain reaction (pt-qPCR) assay enables simultaneous detection of over 100 molecular targets-including bacteria, fungi, viruses, and antimicrobial resistance genes-within 4 h. With its rapid, high-throughput capabilities, this technology facilitates early identification and timely diagnosis of rare pathogen infections. This study retrospectively analyzed one case of pneumonia caused by *C. psittaci* complicated with *H. influenzae* infection, with an emphasis on exploring the role and advantages of pt-qPCR technology in rapid molecular etiological diagnosis of pathogens.

## 2 Materials and methods

### 2.1 The workflow of pt-qPCR

The pt-qPCR was performed using the Broad-Spectrum Pathogen Universal Amplification Kit (Type II, Cat. No. SJ0201, Version V1.1; Hebei Qianye Biotechnology Co., Ltd., Shijiazhuang, China). This qualitative multiplex assay contains 24 reaction groups (BA1–BH3). Each set detects four targets labelled with FAM, VIC, Texas Red, and Cy5 fluorophores, covering more than 100 molecular targets including bacteria, fungi, viruses, and antimicrobial-resistance genes.

Total nucleic acids were extracted from 500 μL of endotracheal aspirate using the GeneRotex 96 automated system (Tianlong Technology, Xi’an, China). Negative and positive controls were processed in parallel for nucleic acid extraction. Preamplification enrichment was performed on a QuantStudio 5 Real-Time PCR System (Thermo Fisher Scientific, Waltham, MA, USA) prior to pt-qPCR, followed by 32 cycles of pt-qPCRamplification. The pt-qPCR thermal profile was: initial denaturation at 95 °C for 5 min; followed by 32 cycles of 95 °C for 10 s and 60 °C for 30 s. Fluorescence signals from each channel were collected upon reaction completion. The cycle threshold (Ct) values were interpreted according to the manufacturer's instructions: Ct ≤ 25, positive; 25 < Ct ≤ 30, weak positive; Ct > 30, gray-zone signal; and No Ct, negative. Of note, due to the pre-amplification enrichment step, the reported Ct values are intended solely for qualitative positive-negative interpretation.

Each run included negative controls, positive controls, and internal controls for each specimen. Valid performance required: no amplification in the negative controls, expected amplification in the positive controls, and successful internal control amplification.

Detailed procedures are provided in Supplementary File S1.

### 2.2 mNGS workflow

According to the standardized protocol for mNGS, an endotracheal aspirate specimen was collected from the patient. The specimen was first liquefied, followed by host-depletion treatment and nucleic acid extraction to obtain the microbial nucleic acid library (see Supplementary File S1). Library preparation was performed following a standardized procedure, including fragmentation, end-repair, A-tailing, UDI adapter ligation, magnetic bead purification, PCR amplification, and a second magnetic bead purification step. Library quality control was conducted using a Qubit 4.0 fluorometer for concentration measurement and a Qsep100 system for fragment size assessment. Sequencing was subsequently performed on the Sikun2000 platform using single-end 75-cycle (SE75) reads.

The raw sequencing data were processed and analyzed through the Autobio in-house bioinformatics pipeline for pathogenic microorganisms, enabling high-resolution taxonomic classification. Multiple filtering strategies were applied to ensure robust and reliable result interpretation. Specifically, fastp (v0.23.0) was used to remove adapters, low-quality reads, and short reads, as well as to perform read deduplication to obtain clean reads. Bowtie2 (v2.3.4.1) was employed to align clean reads to the human reference genome GRCh38.p13 to remove human-derived reads. The remaining non-human reads were classified at the genus level using Kraken2 (v2.1.1), and species-level alignment within each genus was performed using BWA (0.7.17-r1188). The microbial reference database was constructed based on NCBI RefSeq, NCBI GenBank, and the Autobio in-house curated database, covering whole-genome sequences of 17,135 bacteria, 1,597 fungi, 11,807 viruses, and 338 parasites.

Throughout the entire experimental process, positive controls (containing inactivated pathogens) and negative controls (containing human nucleic acids) were included. The negative controls were processed in parallel with clinical specimens through the entire mNGS workflow, including nucleic acid extraction, library preparation, and sequencing. The criterion for a positive result was defined as ≥3 specific reads for a given pathogen, with the additional requirement that the microorganism was not detected in the negative control, or that the read count in the specimen was more than five times higher than that in the negative control, with normal genome coverage.

Finally, all classified sequences were categorized by pathogen type (e.g., bacteria, fungi, viruses, and parasites) and integrated with the patient's clinical data to support etiological interpretation.

## 3 Case description

The patient was a 66-year-old male admitted on November 9, 2025, presenting with a 2-day history of slurred speech and fatigue. He had an 8-year history of hypertension, with a peak blood pressure of 160/100 mmHg; however, he did not take antihypertensive medication regularly or monitor his blood pressure, resulting in poor control. He also had a 10-month history of glomerulonephritis and had been taking traditional Chinese patent medicine orally on a long-term basis without regular renal function follow-up. He denied histories of coronary artery disease and diabetes mellitus. No pharmacological treatment had been administered at the local facility, and his symptoms persisted without relief, leading him to present to our hospital for systematic evaluation and management. (Figure 1)

**Figure 1:**
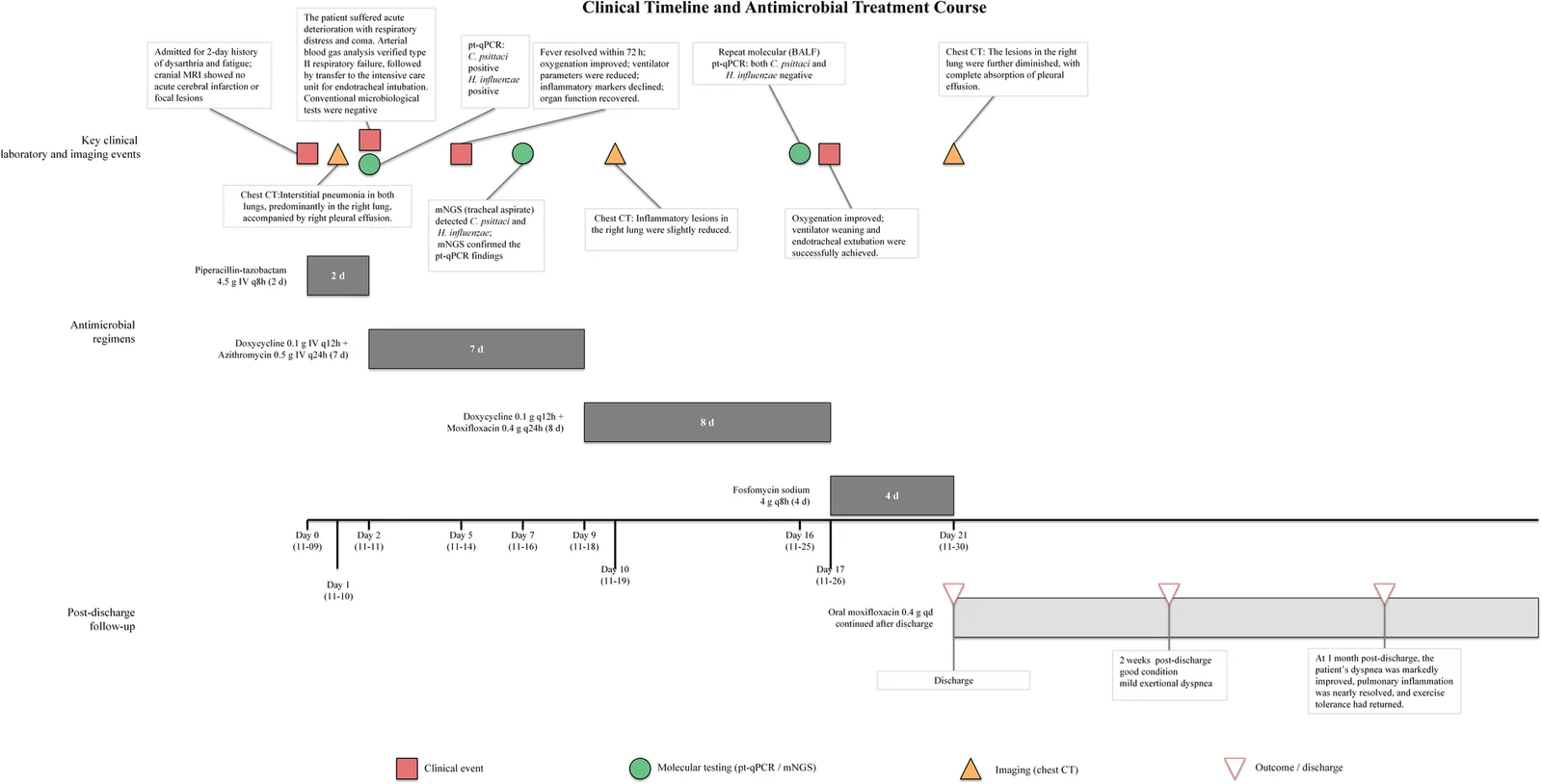
Clinical timeline and antimicrobial treatment course of the patient. The horizontal axis displays calendar dates and corresponding hospital days. Grey horizontal bars represent the duration of different antimicrobial regimens. Clinical events, molecular-imaging findings and clinical outcomes are annotated and aligned to corresponding time points. *C. psittaci*, *Chlamydia psittaci; H. influenzae*, *Haemophilus influenzae*; d, days; IV, intravenous; q8 h, every 8 h; q12 h, every 12 h; q24 h, every 24 h, MRI, Magnetic Resonance Imaging.

On admission, physical examination revealed the following: body temperature 36.8 °C, pulse 92 beats per minute, respiratory rate 20 breaths per minute, and blood pressure 106/83 mmHg. The patient was conscious but demonstrated impaired speech fluency. Coarse breath sounds and moist rales were auscultated bilaterally. Cardiac rhythm was regular, the abdomen was soft, and no lower limb edema was observed. Muscle strength and tone were normal in all four limbs, and no pathological reflexes were elicited.

Laboratory tests revealed a white blood cell count of 7.20 × 10⁹/L, neutrophil count of 6.44 × 10⁹/L, neutrophil percentage of 89.50%, and D-dimer of 2.62 μg/mL (Table 1). Chest computed tomography indicated bilateral interstitial pneumonia, predominantly involving the right lung (Figure 2). The patient was initially diagnosed with cerebral infarction complicated by pulmonary infection, and was treated with cerebral metabolism-promoting agents and intravenous piperacillin-tazobactam sodium at 4.5 g every 8 h for anti-infective therapy. On November 9, cranial magnetic resonance imaging (MRI) revealed no acute cerebral infarction or focal lesions, thereby excluding the possibility of acute cerebrovascular disease.

**Table 1:** Results of laboratory tests of the patient at different times.

Laboratory test	2025								
	Normal range	November 10	November 11	November 14	November 17	November 20	November 23	November 26	November 29
*Blood routine*									
WBC (×109/L)	3.5–9.5	7.2	6.6	7.38	8.37	8.21	5.26	4.68	4.66
NEU%	40–75	89.5	94.8	83.6	80.3	85.6	74.2	68.3	46.6
LYMPH%	20–50	7.1	2.9	10.1	13.9	10.8	20.7	21.5	40.1
*Inflammatory index*									
NLR	NA	12.63	32.95	8.23	5.79	7.89	3.57	3.16	1.16
PLR	NA	250.98	815.79	312	221.55	250.56	235.78	274.26	158.29
SII	NA	1616.31	5106.84	1925.04	1488.83	1758.94	917.18	874.88	343.49
SIRI	NA	3.03	4.94	3.78	2.72	2.21	0.86	1.33	0.56
PCT (ng/mL)	0–0.05	-	58.9	6.12	0.771	0.409	0.19	0.106	0.095
IL-6 (pg/mL)	0–10	-	147.52	152.12	75.9	39.78	38.57	33.83	26.88
D-dimer (ug/mL)	0–0.55	2.62	3.2	6.74	5.32	4.58	2.82	2.49	3.22
FIB (g/L)	2–4	8.49	9	8.49	8.03	6.54	5.94	5.84	4.64
*Biochemical indexes*									
BUN (mmol/L)	1.7–8.3	10.18	17.36	17.59	14.56	11.03	11.22	6.54	6.07
sCr (*μ*mol/L)	57–111	111.1	203.7	101.6	116	97.6	103.7	82	87.7
ALB (g/L)	35–52	31.1	33.4	30	31.2	31.5	32.6	35.5	36.3
PA (mg/L)	170–420	102	107	139	152	198	209	209	247
ALT (U/L)	0–41	31.3	41.3	86.6	61.8	78.9	51	34.3	25.5
AST (U/L)	0–37	67.4	87.9	170.6	68	69.7	43	27.4	18.4

NA, not applicable. These indices are calculated parameters derived from complete blood-count results and have no standardized laboratory reference ranges; −, laboratory data not available because the corresponding tests were not ordered on those dates.

WBC, white blood cell counts; NEU%, neutrophil percentage; LYMPH%, lymphocyte percentage; NLR, neutrophil-to-lymphocyte ratio; PLR, Platelet-to-Lymphocyte Ratio; SII, Systemic Immune-Inflammation Index; SIRI, Systemic Inflammation Response Index；PCT, procalcitonin; IL-6 Interleukin-6; FIB, fibrinogen; BUN, blood urea nitrogen; sCr, serum creatinine; ALB, albumin; PA, prealbumin;ALT, alanine aminotransferase; AST, aspartate aminotransferase.

**Figure 2:**
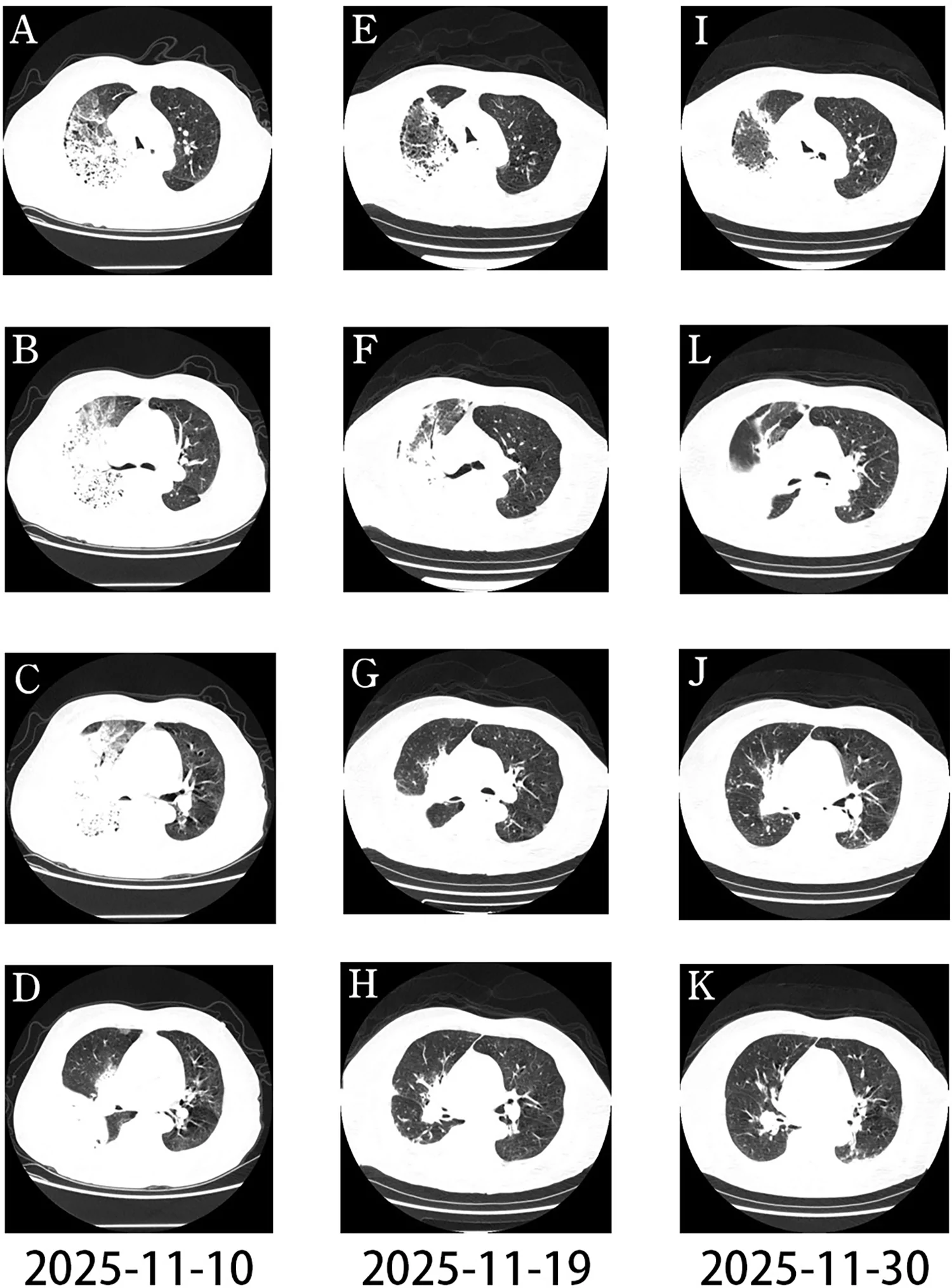
Chest computed tomography at different time points after treatment. Serial axial chest CT images showing dynamic changes in pulmonary lesions at anatomically matched levels across three time-points. Columns represent three serial CT examinations: 2025-11-10 (**A-D**, admission), 2025-11-19 (**E-H**, during antimicrobial therapy), and 2025-11-30 (**I-L**, on the day of hospital discharge). Each horizontal row corresponds to a matched anatomical plane. Panels **A, E, I** are slices obtained at the main pulmonary-artery plane (primary reference landmark). Panels **B, F, J; C, G, K; D, H, L** show sequentially inferior contiguous matched axial slices. (**A-D**) Interstitial pneumonia in both lungs, predominantly in the right lung, accompanied by right-sided pleural effusion. (**E-H**) Inflammatory lesions in the right lung were partially reduced. (**I-L**) The lesions in the right lung were further diminished, with complete resolution of pleural effusion.

On November 11, the patient's condition deteriorated sharply, with high fever (maximum temperature 40.0 °C), tachypnea, dyspnea, and coma. Physical examination revealed comatose consciousness, irregular cardiac rhythm with a heart rate of 110 beats per minute, and blood pressure of 99/62 mmHg. The patient presented with dyspnea and lip cyanosis. Scattered moist rales were audible in both lungs. Peripheral oxygen saturation remained approximately 80% under high-flow mask oxygen. Arterial blood gas analysis showed pH 7.15, PaCO₂ 67 mmHg, and PaO₂ 124 mmHg (oxygen at 6 L/min via face mask), indicating type II respiratory failure. The patient was subsequently transferred to the intensive care unit for endotracheal intubation and mechanical ventilation.

The patient presented with acute onset, characterized by high fever and dyspnea, with rapid disease progression and development of respiratory failure within a short period. Imaging examination revealed bilateral interstitial lung lesions. Empirical treatment with broad-spectrum antibiotics showed poor efficacy. Based on these clinical features, differential diagnoses including viral, bacterial, and atypical pathogen infections were considered. Further history taking revealed a history of long-term pigeon raising, indicating exposure risk to avian-derived pathogens such as *C. psittaci*. Comprehensive etiological screening was performed: respiratory viral nucleic acid testing for severe acute respiratory syndrome coronavirus 2, adenovirus, respiratory syncytial virus, influenza A virus, influenza B virus, human metapneumovirus, and parainfluenza virus were all negative. Sputum culture revealed normal oropharyngeal flora. Fungal (1→3)-β-D-glucan was <37.5 pg/mL (negative), and the galactomannan assay was negative. Conventional testing failed to identify the causative pathogen. On November 11, endotracheal aspirate (ETA) was collected for pt-qPCR testing, and subsequently, the same specimen was subjected to mNGS. The pt-qPCR results obtained on the same day showed Ct values of 21.15 for *C. psittaci* and 12.66 for *H. influenzae* (Figure 3), with both pathogens testing positive. Accordingly, the patient was ultimately diagnosed with severe pneumonia caused by mixed infection with *C. psittaci* and *H. influenzae*. On November 16, mNGS results showed 159,159 sequence reads for *Haemophilus* species and 843 sequence reads for *C. psittaci* (Supplementary Table S1).

**Figure 3:**
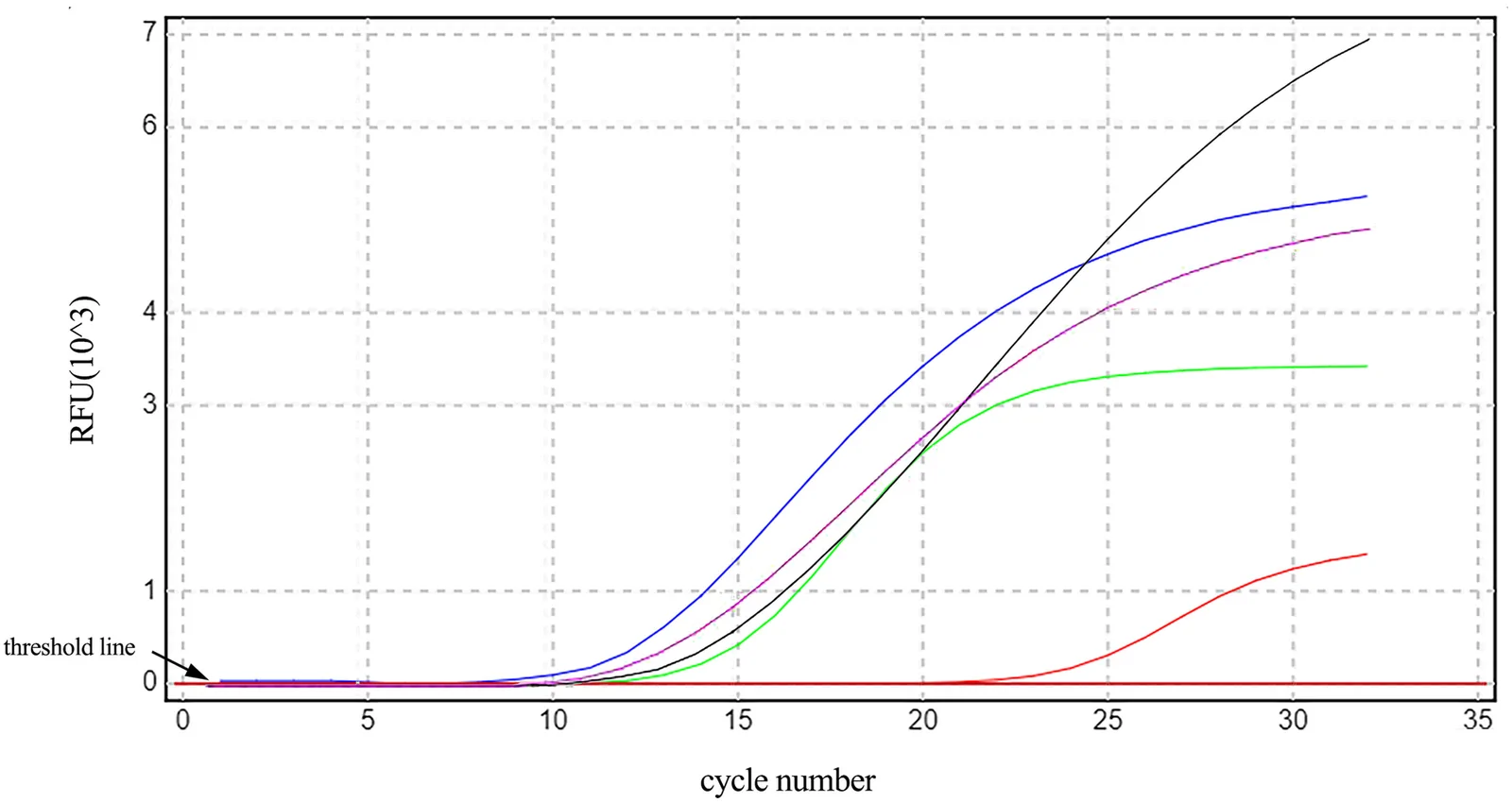
Amplification plots from pt-qPCR assay for the detection of *C. psittaci* and *H. influenzae.* Fluorescence signal (RFU, relative fluorescence units) is plotted against the number of amplification cycles. The horizontal dashed line indicates the threshold for Ct determination (threshold line). Curve assignments are as follows: *H. influenzae* (blue, Ct = 12.66); *C. psittaci* (red, Ct = 21.15); human DNA (internal control, green, Ct = 14.15); positive control for *C. psittaci* (purple, Ct = 12.56); positive control for *H. influenzae* (black, Ct = 13.25); and negative control (maroon, No Ct). The threshold was set automatically by the instrument software. *C. psittaci*, *Chlamydia psittaci*; *H. influenzae*, *Haemophilus influenzae*.

Following confirmation of the diagnosis, a targeted therapeutic regimen was initiated: intravenous doxycycline 0.1 g every 12 h combined with intravenous azithromycin 0.5 g every 24 h. Azithromycin was discontinued after one week, and the regimen was modified to intravenous doxycycline 0.1 g every 12 h combined with intravenous moxifloxacin 0.4 g every 24 h, which was continued thereafter. Concurrent with combination anti-infective therapy, protective lung ventilation strategy, prone positioning, lung recruitment maneuvers, and intermittent fiberoptic bronchoscopy with sputum aspiration and bronchoalveolar lavage were administered. The patient's body temperature gradually returned to normal within 72 h, the oxygenation index increased, and ventilator parameters were progressively reduced. Follow-up laboratory examinations showed gradual decreases in inflammatory markers including procalcitonin (PCT), interleukin-6 (IL-6), systemic immune-inflammation index (SII), and systemic inflammation response index (SIRI), with significant improvement in hepatic and renal function compared with previous results (Table 1). Chest computed tomography was re-examined on November 19 and November 30, respectively, and demonstrated significant absorption of pulmonary inflammatory lesions compared with previous imaging (Figure 2). After two weeks of anti-infective therapy, 10 mL of bronchoalveolar lavage fluid (BALF) was collected via bronchoscopy on November 25 for repeat pt-qPCR testing. Results showed both *C. psittaci* and *H. influenzae* were negative. The patient's clinical symptoms improved further. On November 26, he was successfully weaned from mechanical ventilation and extubated. On the same day, doxycycline and moxifloxacin hydrochloride were discontinued, and fosfomycin was administered to continue anti-infective therapy. After his condition stabilized, he was discharged on November 30. Following discharge, the patient continued taking moxifloxacin (0.4 g once daily). At the 2-week post-discharge follow-up, the patient was in generally good condition, with no chest tightness or discomfort at rest, and mild dyspnea upon exertion. At the 1-month follow-up, the patient's dyspnea symptoms had markedly improved, pulmonary inflammatory lesions were essentially absorbed, and exercise tolerance had returned to the pre-illness state.

## 4 Patient perspective

I am a 66-year-old man with poorly controlled hypertension and glomerulonephritis. On admission, I presented with slurred speech and fatigue. The doctors initially suspected that I might have had a cerebral infarction. However, shortly after admission, my condition deteriorated dramatically. I developed a high fever, fell into a coma, and had to be placed on a ventilator in the intensive care unit. Routine tests failed to identify the cause of my pneumonia. Because I had been raising pigeons for many years, the doctors suspected an avian-related infection. The pt-qPCR quickly confirmed that I had a coinfection with *Chlamydia psittaci* and *Haemophilus influenzae*, which was later validated by mNGS. After targeted antibiotic therapy, my condition gradually improved, and I was eventually discharged. My experience suggests that for patients with relevant exposure history and rapidly progressing pneumonia, rapid molecular tests such as pt-qPCR can play a critical role-helping clinicians achieve timely diagnosis and initiate effective treatment.

## 5 Discussion

*C. psittaci* is an obligate intracellular Gram-negative bacterium and a zoonotic pathogen with a broad host range (6). Birds serve as the natural hosts of *C. psittaci*, and humans can contract psittacosis through direct contact with feces, urine and respiratory secretions of infected birds or poultry (7). After entering the human body via the respiratory tract, *C. psittaci* rapidly divides and proliferates, disseminates through the blood, invades the reticuloendothelial system, and causes pathological changes in the lungs, liver, kidneys, hematological system and neuromuscular system (8). In retrospect, the dysarthria and fatigue observed at admission were considered possible extrapulmonary manifestations of *C. psittaci* infection; however, this interpretation remains speculative, as definitive proof is lacking. The clinical manifestations of psittacosis vary greatly, ranging from common infectious symptoms to severe pneumonia and multisystem involvement induced by infection (9). Most patients with psittacosis pneumonia have a history of exposure to poultry or pigeons. In the present study, the patient had a long-term pigeon-raising history, which served as a key clue for the diagnosis of psittacosis.

*H. influenzae* is a pleomorphic, capnophilic Gram-negative coccobacillus. It is not only an important pathogen for acute diseases such as otitis media and pneumonia, but also a major cause of acute exacerbation of chronic pulmonary diseases (10). This pathogen is mainly transmitted by inhalation of respiratory droplet secretions from infected individuals or through close direct contact (11), while upper respiratory tract colonization is a critical risk factor for human-to-human transmission via saliva droplets (12). Elderly individuals often have multiple underlying diseases and carry a higher risk of invasive bacterial infection (13). Epidemiological data have shown that among the ten major respiratory pathogens detected in adult community-acquired pneumonia (CAP), the detection rate of *H. influenzae* is 10.67%, ranking second (14). However, *H. influenzae* is a common colonizer of the human upper respiratory tract. In this case, the organism was determined to be a true pathogen rather than simple colonization, based on the following evidence. The tested specimens, including deep ETA and BALF, were collected immediately after ICU admission, effectively minimizing contamination by bacteria colonizing the upper respiratory tract (15). mNGS analysis revealed a high relative abundance of this organism, consistent with active pulmonary infection. The patient presented with typical manifestations of severe pneumonia, including high fever and pulmonary consolidation, accompanied by persistently elevated inflammatory markers. Following antimicrobial therapy, his body temperature normalized and inflammatory markers declined significantly, with follow-up pathogen testing yielding negative results. Furthermore, as an elderly male with multiple underlying cerebrovascular diseases, the patient was at high risk for invasive bacterial infection (13). Collectively, these findings exclude simple airway colonization.

It remains unclear whether the patient was initially infected with *C. psittaci* or *H. influenzae*. The patient had a definite history of pigeon exposure, and pigeons were presumed to be the source of *C. psittaci* infection. *C. psittaci* infection can induce the release of pro-inflammatory cytokines such as IL-1, IL-6, and TNF-α, leading to systemic inflammatory response (16). Similarly, NTHi promotes the production of IL-6 and IL-1β through activation of macrophage TLR2/4 and the NLRP3 inflammasome pathway, further exacerbating the inflammatory response (17). The NLR reflects the peripheral inflammatory status and may serve as an effective tool to rapidly evaluate disease severity (18). Following admission, the patient developed progressive multisystem impairment. Laboratory examinations revealed elevated neutrophil ratio, NLR, IL-6, PCT, SII and SIRI, indicating a severe systemic inflammatory response. SIRI is a reliable indicator for assessing inflammatory status, which is consistent with the findings reported by Zhu et al. (19), whereas the clinical significance of elevated SII remains to be further explored. Elevated D-dimer levels suggest a hypercoagulable state and secondary fibrinolytic activation, consistent with coagulation dysfunction associated with severe infection. Elevated D-dimer levels are closely correlated with infectious diseases (20). Elevated ALT and AST indicated liver injury, while decreased ALB suggested hypoalbuminemia and impaired hepatic synthetic function, which further inhibited protein synthesis (21). These findings are in accordance with the previous research results of Xu et al. (22) and Li (23), which may be attributed to reduced albumin intake caused by poor appetite, decreased albumin production due to liver damage, and increased albumin consumption induced by high fever. PCT is an important auxiliary indicator for assessing the severity of *C. psittaci* pneumonia (16).

The clinical manifestations and imaging features of psittacosis are non-specific and easily confused with other pulmonary lesions, bringing difficulties to the early differential diagnosis of mixed infection (24). Conventional detection methods, including serological assays, microbial culture of respiratory specimens, are limited by low sensitivity, narrow pathogen coverage and long turnaround time. In the absence of definitive etiological evidence, clinical decision-making largely relies on physicians’ experience and empirical inference (25). Although empirical treatment without definite pathogen detection results is acceptable at the initiation of therapy (26), delayed administration of effective antibiotics may increase the mortality of severe psittacosis cases (1). Therefore, early identification of pathogenic microorganisms in critically ill patients is essential for timely clinical intervention and appropriate antimicrobial administration (27). PCR-based nucleic acid detection represents the fastest and most specific diagnostic approach. In recent years, pt-qPCR technology has become increasingly mature, enabling simultaneous detection of a wide range of pathogens including bacteria, viruses, yeasts, as well as antibiotic resistance genes, and facilitating rapid clinical diagnosis. In this case, the patient rapidly developed respiratory failure and hepatorenal insufficiency after onset, with negative bacterial culture results. In contrast, pt-qPCR rapidly and accurately identified mixed infection with *C. psittaci* and *H. influenzae*, allowing timely adjustment of the clinical treatment regimen. Unlike traditional culture methods that require several days to yield results, pt-qPCR can provide diagnostic information within hours (28). In our case, pt-qPCR reported results within 4 h. The long turnaround time of conventional assays and complicated diagnostic procedures often lead to delayed diagnosis of mixed infections and rare pathogens, which may increase the risk of poor prognosis (29). Long-term empirical use of broad-spectrum antibiotics may drive the emergence and rising prevalence of antimicrobial resistance among pathogens (30). In this study, mNGS was further applied to verify the pt-qPCR findings. As a novel molecular detection technology, mNGS features high throughput and broad pathogen coverage. It can detect all nucleic acid sequences in clinical specimens in a single sequencing run and accurately identify microbial species simultaneously (31). In the present case, mNGS also detected both *C. psittaci* and *H. influenzae*, which was consistent with pt-qPCR results. The pt-qPCR offers certain advantages in operational simplicity and turnaround time, whereas mNGS has irreplaceable value in broader pathogen coverage and the detection of novel or rare pathogens. The two assays are complementary to each other.

The patient in this case developed rapid respiratory failure and hepatic insufficiency after onset. Early identification of the pathogen and timely adjustment of anti-infective therapy was associated with improvement in organ failure caused by inflammatory responses. After the pathogen was identified by pt-qPCR, the treatment regimen was adjusted accordingly, and the patient's organ dysfunction improved. Tetracyclines are the first-choice agents for *C. psittaci* infection, with macrolides and quinolones as alternative antibacterial drugs (32). Azithromycin belongs to the macrolide class of antibacterial agents and exhibits certain antibacterial activity against *H. influenzae*. Recent global surveillance data show that the resistance rate of *H. influenzae* to macrolides continues to rise, with azithromycin resistance reaching 9.3%–15.3% (33), and exceeding 40% among pediatric isolates in some regions (34). In the early stage of admission, the patient received empirical anti-infective therapy with *β*-lactam antibiotics, which showed poor clinical efficacy. Following pathogen identification by pt-qPCR, the treatment regimen was promptly adjusted to doxycycline combined with azithromycin. However, after 7 days of therapy, the clinical response was assessed as suboptimal by the intensive care unit team; consequently, the regimen was switched to doxycycline combined with moxifloxacin for continued treatment.

This study has several limitations. First, this was a single-case retrospective study that can only reflect the individualized clinical characteristics of this particular mixed infection; therefore, the generalizability of the findings is limited. Second, neither pathogen was subjected to microbial isolation, culture, or strain typing; consequently, their virulence, antimicrobial resistance profiles, and strain characteristics remain unknown. Third, although the combination of multiple clinical indicators, specimen types, and disease progression supports the possibility of *H. influenzae* as a co-pathogen, the possibility of upper respiratory tract colonization cannot be completely excluded. Fourth, despite the patient’s clear history of pigeon exposure, environmental or avian samples were not collected for testing; thus, the source of infection could not be confirmed by molecular epidemiological methods. Fifth, this study only involved clinical efficacy observation without a control group, making it impossible to quantitatively compare the diagnostic performance of pt-qPCR with that of mNGS and conventional testing methods.

## 6 Conclusion

Pneumonia caused by co-infection with *C. psittaci* and *H. influenzae* is rare and presents non-specific clinical manifestations. In this patient with severe pneumonia, avian exposure history, and rapid disease progression, pt-qPCR facilitated early identification of the coinfection and timely adjustment of antimicrobial therapy. This case suggests that rapid targeted multiplex molecular diagnostics may serve as a valuable adjunctive diagnostic tool in patients with severe pneumonia of unknown etiology who have epidemiological risk factors such as avian exposure, particularly when mixed infection is suspected, enabling timely identification of co-pathogens and guiding precision antimicrobial therapy.

## Data Availability

The original contributions presented in the study are included in the article/Supplementary Material, further inquiries can be directed to the corresponding authors.

## References

[1] Xu W Wang Q Li L Zhu B Cai Q Yi X. Case report: metagenomic next-generation sequencing applied in diagnosing psittacosis caused by chlamydia psittaci infection. Front Cell Infect Microbiol. (2023) 13:1249225. 10.3389/fcimb.2023.1249225 37799338 PMC10548267

[2] Ravichandran K Anbazhagan S Karthik K Angappan M Dhayananth B. A comprehensive review on avian chlamydiosis: a neglected zoonotic disease. Trop Anim Health Prod. (2021) 53(4):414. 10.1007/s11250-021-02859-0 34312716 PMC8313243

[3] Hogerwerf L De Gier B Baan B Van Der Heijden W. Chlamydia psittaci (psittacosis) as a cause of community-acquired pneumonia: a systematic review and meta-analysis. Epidemiol Infect. (2017) 145(15):3096–105. 10.1017/s0950268817002060 28946931 PMC9148753

[4] Clinical Microbiology Laboratory Special Committee of Chinese Hospital Association, Infectious Diseases Group of Laboratory Medicine Branch of Chinese Geriatrics Society. (2024). Chinese expert consensus on the diagnosis and treatment of psittacosis. Chin J Clin Infect Dis. (2024) 17:191–204. 10.3760/cma.j.issn.1674-2397.2024.03.002

[5] Yang M Yang D-H Yang H Ding S-Z Liu C-H Yin H-M. Clinical characteristics of chlamydia psittaci pneumonia infection in central south China. Infect Dis Ther. (2022) 11(4):1631–47. 10.1007/s40121-022-00662-4 35723864 PMC9207437

[6] Wang J Wang B Xiao J Chen Y Wang C. Chlamydia psittaci: a zoonotic pathogen causing avian chlamydiosis and psittacosis. Virulence. (2024) 15(1):2428411. 10.1080/21505594.2024.2428411 39541409 PMC11622591

[7] Hogerwerf L Roof I De Jong MJK Dijkstra F Van Der Hoek W. Animal sources for zoonotic transmission of psittacosis: a systematic review. BMC Infect Dis. (2020) 20(1):192. 10.1186/s12879-020-4918-y 32131753 PMC7057575

[8] Qu J Zhang J Chen Y Huang Y Xie Y Zhou M. Aetiology of severe community acquired pneumonia in adults identified by combined detection methods: a multi-centre prospective study in China. Emerg Microbes Infect. (2022) 11(1):556–66. 10.1080/22221751.2022.2035194 35081880 PMC8843176

[9] Cui Z Meng L. Psittacosis pneumonia: diagnosis, treatment and interhuman transmission. Int J Gen Med. (2023) 16:1–6. 10.2147/ijgm.S396074 36628298 PMC9826634

[10] Kappler U Henningham A Nasreen M Yamamoto A Buultjens AH Stinear TP. Tolerance to haemophilus influenzae infection in human epithelial cells: insights from a primary cell-based model. PLoS Pathog. (2024) 20(7):e1012282. 10.1371/journal.ppat.1012282 38990812 PMC11239077

[11] Van Eldere J Slack MP Ladhani S Cripps AW. Non-typeable haemophilus influenzae, an under-recognised pathogen. Lancet Infect Dis. (2014) 14(12):1281–92. 10.1016/s1473-3099(14)70734-0 25012226

[12] Giufrè M Dorrucci M Lo Presti A Farchi F Cardines R Camilli R. Nasopharyngeal carriage of haemophilus influenzae among adults with co-morbidities. Vaccine. (2022) 40(5):826–32. 10.1016/j.vaccine.2021.12.030 34952754

[13] Lemon KP Klepac-Ceraj V Schiffer HK Brodie EL Lynch SV Kolter R. Comparative analyses of the bacterial microbiota of the human nostril and oropharynx. mBio. (2010) 1(3):e00129–10. 10.1128/mBio.00129-10 20802827 PMC2925076

[14] Zhang L Xiao Y Zhang G Li H Zhao J Chen M. Identification of priority pathogens for aetiological diagnosis in adults with community-acquired pneumonia in China: a multicentre prospective study. BMC Infect Dis. (2023) 23(1):231. 10.1186/s12879-023-08166-3 37059987 PMC10103676

[15] Tsai CH Liu LT Hsu EC Sung SY Tsai JJ. Case report: an endogenous endophthalmitis case caused by haemophilus influenzae. Front Med (Lausanne). (2025) 12:1692870. 10.3389/fmed.2025.1692870 41221525 PMC12597914

[16] Chen X Cao K Wei Y Qian Y Liang J Dong D. Metagenomic next-generation sequencing in the diagnosis of severe pneumonias caused by chlamydia psittaci. Infection. (2020) 48(4):535–42. 10.1007/s15010-020-01429-0 32314307 PMC7223968

[17] Huska B Ulanova M. Inflammatory responses to non-typeable haemophilus influenzae clinical isolates from invasive and non-invasive infections. Pathogens. (2025) 14(3):210. 10.3390/pathogens14030210 40137696 PMC11945879

[18] Huang L Weng B Wang M Weng J Du X Ju Y. The improved prediction value of neutrophil to lymphocyte ratio to pneumonia severity scores for mortality in the older people with community-acquired pneumonia. BMC Geriatr. (2025) 25(1):485. 10.1186/s12877-025-06121-2 40604508 PMC12224791

[19] Zhu C Ye T Ye C Xu M He Y Wang D. Clinical characteristics of chlamydia psittaci pneumonia: a single-center, retrospective study over 5 years. BMC Infect Dis. (2025) 25(1):1027. 10.1186/s12879-025-11450-z 40819069 PMC12358061

[20] Meini S Sozio E Bertolino G Sbrana F Ripoli A Pallotto C. D-dimer as biomarker for early prediction of clinical outcomes in patients with severe invasive infections due to streptococcus pneumoniae and neisseria meningitidis. Front Med (Lausanne). (2021) 8:627830. 10.3389/fmed.2021.627830 33937280 PMC8081958

[21] Ehlting C Wolf SD Bode JG. Acute-phase protein synthesis: a key feature of innate immune functions of the liver. Biol Chem. (2021) 402(9):1129–45. 10.1515/hsz-2021-0209 34323429

[22] Xu T Yuan Q Wang J Wu Z Chen Z Wang Z. Clinical characteristics and risk factors associated with severe community-acquired pneumonia infected by chlamydia psittaci. Microbiol Spectr. (2025) 13(11):e0047725. 10.1128/spectrum.00477-25 40990897 PMC12584628

[23] Li X Xiao T Hu P Yan K Wu J Tu X. Clinical, radiological and pathological characteristics of moderate to fulminant psittacosis pneumonia. PLoS One. (2022) 17(7):e0270896. 10.1371/journal.pone.0270896 35816485 PMC9273088

[24] Qin Z Zou Y Huang Z Yu N Deng Z Chen Z. Metagenomic next-generation sequencing contributes to the diagnosis of mixed pulmonary infection: a case report. Ann Clin Microbiol Antimicrob. (2022) 21(1):52. 10.1186/s12941-022-00545-z 36434704 PMC9701064

[25] Lhommet C Garot D Grammatico-Guillon L Jourdannaud C Asfar P Faisy C. Predicting the microbial cause of community-acquired pneumonia: can physicians or a data-driven method differentiate viral from bacterial pneumonia at patient presentation? BMC Pulm Med. (2020) 20(1):62. 10.1186/s12890-020-1089-y 32143620 PMC7060632

[26] Li Y Lei J Ren Z Ma X. Case report: metagenomic next-generation sequencing assists in dynamic pathogen monitoring: powerful tool for progressing severe pneumonia. Front Cell Infect Microbiol. (2023) 13:1230813. 10.3389/fcimb.2023.1230813 37743869 PMC10512180

[27] Zhao Y Zhang W Zhang X. Application of metagenomic next-generation sequencing in the diagnosis of infectious diseases. Front Cell Infect Microbiol. (2024) 14:1458316. 10.3389/fcimb.2024.1458316 39619659 PMC11604630

[28] Serapide F Pallone R Quirino A Marascio N Barreca GS Davoli C. Impact of multiplex pcr on diagnosis of bacterial and fungal infections and choice of appropriate antimicrobial therapy. Diagnostics (Basel). (2025) 15(8):1044. 10.3390/diagnostics15081044 40310414 PMC12026191

[29] Garrido P Gabaldó-Barrios X Pujol-Bajador I Fernández L Ballester F Garrido R. Assessing the utility of multiplexed polymerase chain reaction in detecting microorganisms causing infections in critically ill patients. Curr Microbiol. (2023) 80(11):348. 10.1007/s00284-023-03461-3 37733061 PMC10514122

[30] Wei J Uppal A Nganjimi C Warr H Ibrahim Y Gu Q. No evidence of difference in mortality with amoxicillin versus co-amoxiclav for hospital treatment of community-acquired pneumonia. J Infect. (2024) 88(6):106161. 10.1016/j.jinf.2024.106161 38663754 PMC11893475

[31] Ge M Gan M Yan K Xiao F Yang L Wu B. Combining metagenomic sequencing with whole exome sequencing to optimize clinical strategies in neonates with a suspected central nervous system infection. Front Cell Infect Microbiol. (2021) 11:671109. 10.3389/fcimb.2021.671109 34222042 PMC8253254

[32] Balsamo G Maxted AM Midla JW Murphy JM Wohrle R Edling TM. Compendium of measures to control chlamydia psittaci infection among humans (psittacosis) and pet birds (avian chlamydiosis), 2017. J Avian Med Surg. (2017) 31(3):262–82. 10.1647/217-265 28891690

[33] Ahmad I Kubaev A Zwamel AH R. R Baldaniya L Kaur J. Insights into haemophilus macrolide resistance: a comprehensive systematic review and meta-analysis. PLoS Negl Trop Dis. (2025) 19(3):e0012878. 10.1371/journal.pntd.0012878 40036252 PMC11902202

[34] Abavisani M Keikha M Karbalaei M. First global report about the prevalence of multi-drug resistant haemophilus influenzae: a systematic review and meta-analysis. BMC Infect Dis. (2024) 24(1):90. 10.1186/s12879-023-08930-5 38225571 PMC10789054

