# Supplementary material for: Pathogen-targeted quick multiplex PCR identification of Chlamydia psittaci and Haemophilus influenzae coinfection in pneumonia: a case report
Source: Front Med (Lausanne). 2026 Oct 5;13:1890644. doi: 10.3389/fmed.2026.1890644 (PMC13677695; doi:10.3389/fmed.2026.1890644)
Supplement: Supplementary file 1 [file Datasheet1.pdf]

CARE Checklist of information to include when writing a case report

CARE case report guidelines

(cc) BY-NC-ND

Topic	Item	Checklist item description	Reported on Line
Title	1	The diagnosis or intervention of primary focus followed by the words "case report"	Title
Key Words	2	2 to 5 key words that identify diagnoses or interventions in this case report, including "case report"	12-13
Abstract (no references)	3a	Introduction: What is unique about this case and what does it add to the scientific literature?	17-18,26-28
	3b	Main symptoms and/or important clinical findings	18-21
	3c	The main diagnoses, therapeutic interventions, and outcomes	21-26
	3d	Conclusion—What is the main "take-away" lesson(s) from this case?	26-28
Introduction	4	One or two paragraphs summarizing why this case is unique (**may include references**)	29-60
Patient Information	5a	De-identified patient specific information	94
	5b	Primary concerns and symptoms of the patient	94-95
	5c	Medical, family, and psycho-social history including relevant genetic information	95-96
	5d	Relevant past interventions with outcomes	-
Clinical Findings	6	Describe significant physical examination (PE) and important clinical findings	97-102
Timeline	7	Historical and current information from this episode of care organized as a timeline	96
Diagnostic Assessment	8a	Diagnostic testing (such as PE, laboratory testing, imaging, surveys)	103-105,126-132
	8b	Diagnostic challenges (such as access to testing, financial, or cultural)	222-226
	8c	Diagnosis (including other diagnoses considered)	106,134-135
	8d	Prognosis (such as staging in oncology) where applicable	159-162
Therapeutic Intervention	9a	Types of therapeutic intervention (such as pharmacologic, surgical, preventive, self-care)	107-108,115-116,138-144,155-158
	9b	Administration of therapeutic intervention (such as dosage, strength, duration)	108,139-142,158
	9c	Changes in therapeutic intervention (with rationale)	140-142,155-158,258-268
Follow-up and Outcomes	10a	Clinician and patient-assessed outcomes (if available)	154-162
	10b	Important follow-up diagnostic and other test results	146-154,160-162
	10c	Intervention adherence and tolerability (How was this assessed?)	159-162
	10d	Adverse and unanticipated events	159-162
Discussion	11a	A scientific discussion of the strengths AND limitations associated with this case report	264-268,269-280
	11b	Discussion of the relevant medical literature **with references**	163-280
	11c	The scientific rationale for any conclusions (including assessment of possible causes)	109,131-132
	11d	The primary "take-away" lessons of this case report (without references) in a one paragraph conclusion	285-288
Patient Perspective	12	The patient should share their perspective in one to two paragraphs on the treatment(s) they received	-
Informed Consent	13	Did the patient give informed consent? Please provide if requested	Yes ✓ No
