# Supplementary material for: Pathogen-targeted quick multiplex PCR identification of Chlamydia psittaci and Haemophilus influenzae coinfection in pneumonia: a case report
Source: Front Med (Lausanne). 2026 Oct 5;13:1890644. doi: 10.3389/fmed.2026.1890644 (PMC13677695; doi:10.3389/fmed.2026.1890644)
Supplement: Supplementary file 2 [file Supplementaryfile1.docx]

Supplementary Material

# 1 Supplementary File S1: Detailed experimental procedures for pathogen‑targeted quick multiplex PCR

## 1.1 Assay kit overview

Pathogen‑targeted quick multiplex PCR (pt‑qPCR) was performed using the Broad‑Spectrum Pathogen Universal Amplification Kit (Type II, Cat. No. SJ0201, Version V1.1; Hebei Qianye Biotechnology Co., Ltd., Shijiazhuang, China). This commercial qualitative kit contains 24 sets of multiplex qPCR reagents (BA1‑BH3). Each set detects four targets labelled with FAM, VIC, Texas Red, and Cy5 fluorophores, covering more than 100 molecular targets including bacteria, fungi, viruses, and antimicrobial‑resistance genes. All primer‑probe sets are designed against species‑specific conserved sequences. Detailed information on pathogens and corresponding fluorescence channels for each reaction group is provided in the manufacturer’s official manual.

BA1-BH3: Specific Multiplex qPCR Reagents, the corresponding target and fluorescent channel details are as follows:

| **Group** | **FAM** | **VIC** | **Texas Red** | **Cy5** |
| --- | --- | --- | --- | --- |
| BA1 | *Streptococcus pneumoniae* | *Haemophilus influenzae* | *Brucella* spp. | *Moraxella catarrhalis* |
| BB1 | *Bordetella pertussis* | *Whippleiella whipplei* | *Legionella pneumophila* | *Corynebacterium diphtheriae* |
| BC1 | *Mycoplasma pneumoniae* | *Human bocavirus* | *Chlamydia pneumoniae* | *Chlamydia psittaci* |
| BD1 | *Mycobacterium avium* | *Mycobacterium intracellulare** | *Mycobacterium abscessus* | RNA Internal Control |
| BE1 | *Talaromyces marneffei* | *Mycobacterium tuberculosis* complex | *Aspergillus nidulans* / *Aspergillus versicolor* / *Aspergillus oryzae* | *Mycobacterium tuberculosis* |
| BF1 | *Nocardia otitidiscaviarum* | *Nocardia gelsenkirchensis* | *Nocardia abscessus* | *Nocardia terpenica* |
| BG1 | *Trichosporon asahii* | *Candida tropicalis* | *Candida glabrata* | *Candida albicans* |
| BH1 | *Aspergillus niger* | *Aspergillus terreus* | *Aspergillus flavus* | *Aspergillus fumigatus* |
| BA2 | *Cryptococcus neoformans* | *SARS‑CoV‑2* | *Parainfluenza virus** | *Pneumocystis jirovecii* |
| BB2 | *Candida parapsilosis* | *Candida lusitaniae* | *Candida krusei* | *Candida dubliniensis* |
| BC2 | *Enterococcus avium* | *Enterococcus faecalis* | *Enterococcus faecium* | *Serratia* spp. |
| BD2 | *Staphylococcus aureus* | DNA Internal Control | *Salmonella* spp. | *Burkholderia gladioli* |
| BE2 | *Klebsiella pneumoniae* | *Pseudomonas aeruginosa* | *Stenotrophomonas maltophilia* | *Acinetobacter baumannii* |
| BF2 | *Burkholderia cepacia* complex | *Escherichia coli* | *Enterobacter cloacae* | *Listeria monocytogenes* |
| BG2 | *Human herpesvirus 1* | *Cytomegalovirus* | *Epstein‑Barr virus* | *Varicella‑zoster virus* |
| BH2 | *Treponema pallidum* | *Ureaplasma urealyticum* | *Chlamydia trachomatis* | *Mycoplasma hominis* |
| *BA3* | *Adenovirus* | *Enterovirus 71* | *Severe fever with thrombocytopenia syndrome virus* | *Coxsackievirus A16* |
| BB3 | *Streptococcus suis* | *Streptococcus pyogenes* | *Proteus mirabilis* | *Klebsiella aerogenes* / *Klebsiella oxytoca* |
| BC3 | *Influenza A virus* | *Influenza B virus* | *Echovirus* | *Respiratory syncytial virus** |
| BD3 | *Human rhinovirus* | *Human metapneumovirus* |  | *Human classical coronavirus** |
| BE3 | *Orientia tsutsugamushi* | *Leptospira interrogans* | *Coxiella burnetii* | *Rickettsia felis* |
| BF3 | *Mycobacterium scrofulaceum* | *Mycobacterium kansasii* | *Rhizopus oryzae* | *Mycobacterium fortuitum* |
| BG3 | MecA | KPC | VanA | VIM |
| BH3 | NDM | ampC | IMP | MCR‑1 |

**Notes:**

1. *Mycobacterium intracellulare* includes *Mycobacterium intracellulare* and *Mycobacterium paraintracellulare*.
2. Respiratory syncytial virus includes respiratory syncytial virus type A and type B.
3. Parainfluenza virus includes parainfluenza virus type 1, type 2, type 3 and type 4.
4. Human classical coronavirus includes human coronavirus 229E, NL63, HKU1 and OC43.

## 1.2 Specimen pretreatment and nucleic acid extraction

For endotracheal aspirate specimens, 500 μL sample was predigested with 50 μL of 0.5 M dithiothreitol (DTT) and incubated at room temperature for 10 min. The processed sample was transferred to a lysis tube, supplemented with 20 μL lyticase and incubated at 25 °C for 20 min, followed by mechanical bead‑beating at 70 Hz for 120 s over 5 cycles. After centrifugation, total nucleic acids were purified using the GeneRotex 96 automated nucleic‑acid extraction system (Tianlong Science and Technology, Xi’an, China). Negative controls, positive controls and clinical test samples were processed for nucleic‑acid extraction in parallel.

## 1.3 Preamplification enrichment

Preamplification enrichment was performed prior to multiplex qPCR. The enrichment reaction system consisted of 7 μL total nucleic‑acid template, 28 μL kit enrichment reagent FJ, and 40 μL protective reagent OL. After brief centrifugation, the mixture was subjected to preamplification on a QuantStudio 5 Real‑Time PCR instrument (Thermo Fisher Scientific, Waltham, MA, USA) with the following thermal profile: 55 °C for 15 min; 95 °C for 30 s; 10 cycles of 95 °C for 10 s and 63 °C for 1 min; final extension at 72 °C for 5 min; and hold at 4 °C.

## 1.4 Multiplex qPCR amplification

One microliter of enriched product was mixed with 9 μL of each BA1‑BH3 multiplex qPCR reagent and loaded onto the PCR microplate following the preset layout. The plate was sealed with adhesive film and briefly centrifuged before amplification. The qPCR thermal profile was: 95 °C for 5 min; 32 cycles of 95 °C for 10 s and 60 °C for 30 s, with fluorescence signals collected at 60 °C. Amplification was performed on the QuantStudio 5 Real‑Time PCR instrument.

## 1.5 Interpretation criteria for amplification results

Fluorescence signals from each channel were collected upon reaction completion. Result interpretation combined thresholds from the kit manual (V1.1) Per the manufacturer’s manual: Ct ≤ 25 was defined as positive; 25 < Ct ≤ 30 as weak positive; Ct > 30 as grey‑zone signal; and NoCt as negative.

Of note, this assay includes a pre‑amplification enrichment step. Reported Ct values are intended solely for qualitative positive‑negative interpretation.

## 1.6 Quality‑control measures

This assay incorporates an RNA internal control (BD1 reaction group) and a DNA internal control (BD2 reaction group) to monitor nucleic‑acid extraction integrity and detect PCR‑inhibitory substances. Each experimental batch contained extraction negative control, reagent no‑template control, and kit‑supplied positive control. Valid performance required no amplification signal in negative controls and expected target amplification within acceptable Ct ranges for the positive control. Internal amplification controls (RNA and DNA internal references) were tested for every clinical specimen. Failure of internal‑control amplification indicated extraction failure or PCR inhibitors and rendered corresponding sample results invalid.

## 1.7 Local performance verification

Our laboratory completed local performance verification of this assay according to CNAS‑GL039 and GB/T 37871‑2019. The negative reference compliance rate was 100%; positive clinical specimens showed 100 % concordance with the reference platform; intra‑assay precision was acceptable with all targets CV < 5%; and representative strains were reliably detected near the manufacturer‑claimed limit of detection (1000 copies/mL).

# 2 Supplementary File S2: Step‑by‑step operating procedure for metagenomic next‑generation sequencing (mNGS)

## 2.1 Table 1 Instruments and equipment

| **Instrument** | **Model** | **Manufacturer** |
| --- | --- | --- |
| Vortex mixer | XH‑A | Wuxi Jiarui’an |
| Mini centrifuge | YK‑MINI‑7K | Yika (Shanghai) Technology |
| High‑speed centrifuge | Centrifuge 5425 | Eppendorf, Germany |
| Dry‑bath incubator | IB‑10HL | Hangzhou Aosheng Instrument Co., Ltd. |
| PCR amplifier | Veriti | Thermo Fisher Scientific |
| Qubit fluorometer | Qubit® 4.0 | Thermo Fisher Scientific |
| Qsep100 system | Qsep100 | Houze Biotechnology |
| Next‑generation sequencer | Sikun2000 | Zhengzhou Sikun Biological Engineering Co., Ltd. |

## 2.2 Specimen pre‑treatment

**Liquefaction of viscous sputum specimens**: Transfer 750 μL sample into a 2 mL centrifuge tube and add 750 μL sample dilution buffer. Vortex‑mix and incubate at room temperature for 10 min. Gently mix the sample every 2–3 min to achieve sufficient liquefaction of viscous specimens, followed by host‑depletion processing.

**Host‑depletion procedure**: Transfer 1 mL liquefied sample into a 1.5 mL centrifuge tube; supplement to 1 mL with 1×PBS if the sample volume is insufficient. Centrifuge at 12 000 rpm for 10 min and discard 960 μL of the supernatant. Sequentially add 180 μL reaction solution, 10 μL buffer, and 5 μL enzyme solution. Vortex‑mix and perform brief centrifugation, then incubate at 37 °C in a dry‑bath incubator for 20 min with mixing every 10 min. After incubation, briefly centrifuge and add 10 μL stop solution. Mix and centrifuge, then incubate at 70 °C in the dry‑bath incubator for 5 min, and cool down to room temperature for 3 min. Centrifuge at 12 000 rpm for 5 min, discard the supernatant, and resuspend the pellet in 200 μL 1×PBS.

## 2.3 Nucleic‑acid extraction

Add 50 μL lysis enzyme solution to 200 μL host‑depleted sample, vortex‑mix, and incubate at 37 °C for 20 min with mixing every 5 min. Add 20 μL Proteinase K and 250 μL lysis buffer, vortex‑mix, and incubate at 56 °C for 10 min with mixing twice during incubation. Add 250 μL isopropanol and 20 μL magnetic beads, and mix on a vertical mixer at 50 rpm for 6 min. After brief centrifugation, place the tube on a magnetic rack for 1 min and discard the supernatant. Add 500 μL Wash Buffer I, mix vigorously for 1 min, perform brief centrifugation, place on the magnetic rack for 1 min, and discard the supernatant. Add 500 μL Wash Buffer II, mix vigorously for 1 min, perform brief centrifugation, place on the magnetic rack for 1 min, and discard the supernatant; repeat this wash step twice. Keep the tube on the magnetic rack and air‑dry magnetic beads at room temperature until no obvious surface gloss is observed. Add 50 μL elution buffer, vortex‑mix, and incubate at 56 °C for 5 min with gentle shaking to promote sufficient nucleic‑acid elution. Place the tube on the magnetic rack for 1 min, and transfer the supernatant to a new centrifuge tube after complete magnetic‑bead adsorption.

## 2.4 Library preparation

## 2.4.1Fragmentation and A‑tailing

Prepare the reaction system on ice in 0.2 mL PCR tubes:

| **Component** | **Volume per reaction (μL)** |
| --- | --- |
| Sample DNA | X |
| Nuclease‑free water | 50- X |
| Fragmentation, end‑repair and A‑tailing master mix | 10 |
| Total volume | 60 |

For DNA samples with concentration ≥ 0.1 ng/μL, 5 ng DNA was used as input. For samples with DNA concentration ＜ 0.1 ng/μL, the full 50 μL eluate was used.

Mix the prepared reaction system and perform brief centrifugation. Run the thermal profile on a PCR instrument (hot‑lid set to 105 °C):

| **Temperature** | **Duration** |
| --- | --- |
| 4 °C | 1 min |
| 30 °C | 5 min |
| 72 °C | 5 min |
| 4 °C | Hold |

Immediately place PCR tubes on ice after the reaction for subsequent adapter ligation.

**2.4.2 Adapter ligation**

Prepare the ligation system on ice in 0.2 mL PCR tubes:

| **Component** | **Volume per reaction (μL)** |
| --- | --- |
| Fragmented and A‑tailed product | 60 |
| UDI adapter | 5 |
| Ligation buffer | 30 |
| Ligase | 5 |
| Total volume | 100 |

Mix the system and perform brief centrifugation. Run the program on a PCR instrument (hot‑lid turned off):

| **Temperature** | **Duration** |
| --- | --- |
| 20 °C | 15 min |
| 4 °C | Hold |

**2.4.3 Purification of adapter‑ligated products**

Take out the nucleic‑acid purification reagent and equilibrate it to room temperature for 30 min; vortex thoroughly before use. Add 60 μL nucleic‑acid purification reagent to 100 μL ligation product, vortex‑mix, and incubate at room temperature for 5 min. Perform brief centrifugation, place the PCR tube on a magnetic rack and incubate for 5 min until magnetic beads aggregate and the solution becomes clear, then discard the supernatant. Slowly add 200 μL freshly‑prepared 80% ethanol along the tube wall, incubate for 30 s and discard the supernatant; repeat twice. Perform brief centrifugation, place the tube back on the magnetic rack and remove residual ethanol. Open the lid and air‑dry at room temperature for 3–5 min until magnetic beads lose surface gloss. Add 22 μL buffer, vortex‑mix, and incubate at room temperature for 5 min. Perform brief centrifugation, place the tube on the magnetic rack, and transfer 20 μL supernatant to a new PCR tube after bead aggregation.

**2.4.4 PCR amplification**

Prepare the amplification system on ice in 0.2 mL PCR tubes:

| **Component** | **Volume per reaction (μL)** |
| --- | --- |
| Purified adapter‑ligated product | 20 |
| Amplification master mix | 25 |
| Amplification primers | 5 |
| Total volume | 50 |

Mix the system and perform brief centrifugation. Run the thermal profile on a PCR instrument (hot‑lid 105 °C):

| **Temperature** | **Time** | **Cycle number** |
| --- | --- | --- |
| 98 °C | 1 min | 1 |
| 98 °C | 10 s | 14 |
| 60 °C | 30 s |  |
| 72 °C | 30 s |  |
| 72 °C | 5 min | 1 |
| 4 °C | Hold | 1 |

### 2.4.5 Magnetic‑bead purification

Add 45 μL nucleic‑acid purification reagent to 50 μL amplified product, vortex‑mix and incubate at room temperature for 5 min. Perform brief centrifugation, place the PCR tube on a magnetic rack and incubate for 5 min until beads aggregate and the solution turns clear, then discard the supernatant. Slowly add 200 μL freshly‑prepared 80% ethanol along the tube wall, incubate for 30 s and discard the supernatant; repeat twice. Perform brief centrifugation, place the tube back on the magnetic rack and remove residual ethanol. Open the lid and air‑dry at room temperature for 3–5 min until magnetic beads lose surface gloss. Add 22 μL buffer, vortex‑mix and incubate at room temperature for 5 min. Perform brief centrifugation, place the tube on the magnetic rack, and transfer 20 μL supernatant to a new 1.5 mL centrifuge tube after bead aggregation.

## 2.5 Library quality control

Library concentration was quantified using a Qubit fluorometer with the 1× dsDNA HS Assay Kit. Libraries with concentration ≥ 1 ng/μL were considered qualified; unqualified libraries required repeated nucleic‑acid extraction and library construction. Library fragment‑size distribution was evaluated on the Qsep100 system with matching reagents; the expected main fragment peak was approximately 250 bp.

## 2.6 Sequencing

Thaw the sequencing kit and sequencing chip at room temperature in advance. Qualified libraries were pooled by equal‑mass mixing. Transfer 5 μL pooled library into a 1.5 mL centrifuge tube, add 5 μL 0.2 mol/L NaOH, vortex‑mix, briefly centrifuge, and denature at room temperature for 5 min. Immediately add 990 μL library dilution buffer and vortex‑mix after denaturation. Transfer 120 μL denatured library into a 2 mL sterile EP tube, add 1380 μL library dilution buffer, vortex‑mix and briefly centrifuge. Load the total 1500 μL mixture into the sample‑loading well of the sequencing kit. Add the mixture slowly to avoid bubble formation. Inspect for bubbles after loading; tap the sequencing kit gently on the bench to release trapped bubbles if present. Sequencing was performed on the Sikun2000 sequencer with single‑end 75‑ cycle (SE75) mode.

## 2.7 Data analysis

Data analysis was performed using the Autobio Diagnostics pathogenic microbial sequencing analysis software. Briefly, fastp (v0.23.0) was used to remove adapters, low‑quality reads and short‑length reads, and deduplication was performed to generate clean reads. Bowtie2 (v2.3.4.1) was applied to map clean reads against the human reference genome GRCh38.p13 for removal of human‑origin reads. The microbial reference database was constructed based on NCBI RefSeq, NCBI GenBank and the in‑house curated database of Autobio Diagnostics, covering whole‑genome sequences of 17 135 bacteria, 1 597 fungi, 11 807 viruses and 338 parasites. Non‑human reads were preliminarily classified at the genus level using Kraken 2 (v2.1.1). BWA (0.7.17‑r1188) was used for precise species‑level alignment of intra‑genus reads. Proprietary algorithms from Autobio Diagnostics were adopted to extract species‑specific unique reads, and pathogen reports were generated via an in‑house interpretation workflow. Each sequencing run included positive controls containing inactivated pathogens and human‑derived negative controls. A microbial taxon was defined as positive when species‑specific unique reads ≥ 3, together with the following criteria: the target microbe was absent in the negative control or unique reads in the clinical specimen were ≥ 5‑fold higher than those in the negative control, normal genome coverage, and published evidence linking the detected pathogen to infectious diseases at the sampled anatomical site.Relative abundance for each taxon was calculated as the percentage of species‑specific unique reads assigned to that taxon divided by total non‑human microbial reads of the sample.

# 3 Supplementary Table S1: The results of mNGS tests of the patient

Table S1 The results of mNGS tests of the patient

| Type | Results | Reads | Relative abundance ^a^ |
| --- | --- | --- | --- |
| Endotracheal aspirate | *Haemophilus* | 159,159 ^b^ | 50.74% |
| Endotracheal aspirate | *Chlamydia psittaci* | 843 | 0.27% |
| Negative control ^c^ | No detected | 0 | 0% |

a. Relative abundance: the proportion of reads assigned to the genus of the pathogen among all microorganisms of the same category detected.

b. Of the 159,159 reads assigned to the genus *Haemophilus*, 144,330 (90.7%) were specifically identified as *Haemophilus influenzae* at the species level.

c. Negative controls (nuclease-free water) were processed in parallel with clinical specimens through the entire mNGS workflow. No reads for *Chlamydia psittaci* or *Haemophilus* were detected in the negative controls.
